# Supplementary material for: Effectiveness of Nootropics in Combination with Cholinesterase Inhibitors on Cognitive Function in Mild-to-Moderate Dementia: A Study Using Real-World Data
Source: J Clin Med. 2022 Aug 9;11(16):4661. doi: 10.3390/jcm11164661 (PMC9409895; doi:10.3390/jcm11164661)
Supplement: Supplementary file 1 [file jcm-11-04661-s001.zip › Supplementary Table S1.pdf]

Supplementary Table S1. Comparison of the distribution of the three types of cholinesterase inhibitor in the cholinesterase inhibitor only group and the cholinesterase inhibitor and nootropics combination group.

	ChEI only (n = 410)	ChEI + nootropics (n = 173)	$\chi 2$	*p* value
Donepezil	353 (86.1)	153 (88.4)	0.582	0.446
Rivastigmine	93 (22.7)	33 (19.1)	0.935	0.334
Galantamine	28 (6.8)	19 (11.0)	2.832	0.092

Values are presented as number (%).
ChEI, cholinesterase inhibitor.
If subjects were prescribed multiple types of cholinesterase inhibitor, each of them was separately included in each types of cholinesterase inhibitor.
