# Supplementary material for: Effectiveness of Nootropics in Combination with Cholinesterase Inhibitors on Cognitive Function in Mild-to-Moderate Dementia: A Study Using Real-World Data
Source: J Clin Med. 2022 Aug 9;11(16):4661. doi: 10.3390/jcm11164661 (PMC9409895; doi:10.3390/jcm11164661)
Supplement: Supplementary file 1 [file jcm-11-04661-s001.zip › Supplementary Table S2.pdf]

Supplementary Table S2. Results of ANOVA for mini-mental state examination (MMSE) total score and its six-subscale scores among the three cholinesterase and nootropics combination subgroups in Alzheimer's dementia.

MMSE	Choline alfoscerate (A) (n = 74)	Ginkgo biloba (B) (n =39)	Others[1] (C) (n = 21)	F	*p* value	Post-hoc analysis[2]
Total	-0.28 ± 2.57	-0.51 ± 2.82	-2.81 ± 5.15	5.35	0.006	A > C, B > C
Orientation	-0.27 ± 1.87	-0.44 ± 1.83	-1.10 ± 2.17	1.53	0.220	
Immediate recall	-0.08 ± 0.54	0.00 ± 0.32	-0.29 ± 1.10	1.48	0.231	
Attention & calculation	-0.18 ± 1.36	-0.13 ± 1.17	0.14 ± 1.20	0.51	0.603	
Delayed recall	-0.05 ± 1.20	-0.31 ± 1.10	-0.48 ± 0.98	1.39	0.254	
Language	0.22 ± 1.21	0.26 ± 1.21	-0.95 ± 1.86	7.04	0.001	A > C, B > C
Visuospatial construction	0.08 ± 0.61	0.10 ± 0.55	-0.14 ± 0.48	1.438	0.241	

Values are presented as mean ± standard deviation.
MMSE, mini-mental state examination.
[1]Others include acetyl-L-carnitine (n = 10), nicergoline (n = 10), and oxiracetam (n = 1).
[2]Significant group differences ($p < 0.05$) were conducted via Bonferroni multiple comparisons test.
